# Sphingosine-1-phosphate Treatment Improves Cryopreservation Efficiency in Human Mesenchymal Stem Cells

**DOI:** 10.3390/life13061286

**Published:** 2023-05-30

**Authors:** Seong-Ju Oh, Chan-Hee Jo, Tae-Seok Kim, Chae-Yeon Hong, Sung-Lim Lee, Young-Hoon Kang, Gyu-Jin Rho

**Affiliations:** 1 Department of Theriogenology and Biotechnology, College of Veterinary Medicine, Gyeongsang National University, Jinju 52828, Republic of Korea; osj414@gnu.ac.kr (S.-J.O.);; 2 Department of Dentistry, Gyeongsang National University Changwon Hospital, Changwon 51472, Republic of Korea; 3 Department of Dentistry, Institute of Health Sciences, School of Medicine, Gyeongsang National University, Jinju 52727, Republic of Korea

**Keywords:** sphingosine-1-phosphate, actin cytoskeleton, dental pulp-derived mesenchymal stem cells, cryopreservation

## Abstract

The actin cytoskeleton plays a crucial role not only in maintaining cell shape and viability but also in homing/engraftment properties of mesenchymal stem cells (MSCs), a valuable source of cell therapy. Therefore, during the cryopreservation process of MSCs, protecting the actin cytoskeleton from the freezing/thawing stress is critical in maintaining their functionality and therapeutic potential. In this study, the safety and cryoprotective potential of sphingosine-1-phosphate (S1P), which has a stabilizing effect on actin cytoskeleton, on dental pulp-derived MSCs (DP-MSCs) was investigated. Our results demonstrated that S1P treatment did not adversely affect viability and stemness of DP-MSCs. Furthermore, S1P pretreatment enhanced cell viability and proliferation properties of post-freeze/thaw DP-MSCs, protecting them against damage to the actin cytoskeleton and adhesion ability as well. These findings suggest that a new cryopreservation method using S1P pretreatment can enhance the overall quality of cryopreserved MSCs by stabilizing the actin cytoskeleton and making them more suitable for various applications in regenerative medicine and cell therapy.

## 1. Introduction

Mesenchymal stem cells (MSCs) have emerged as ideal candidates in regenerative medicine due to their capability of self-renewal, multi-lineage differentiation, and immunomodulatory properties [1,2]. Among MSCs isolated from various tissues, dental pulp-derived MSCs (DP-MSCs) have shown great potential for cell-based therapies due to their readily accessibility from discarded teeth without invasive surgical procedures [3,4,5].

In order to obtain sufficient cell numbers for therapeutic applications, in vitro expansion of MSCs is necessary. However, freshly isolated MSCs have a limited number and may not produce enough cells within a short time frame [6]. Cryopreservation is therefore a reliable technology for the long-term storage of MSCs, ensuring their availability. Slow freezing, cooling at a ramp rate of −1 °C/min to −80 °C, and storing in liquid nitrogen (−196 °C) is a conventional method for cell cryopreservation. Upon cryopreservation at −196 °C cryogenic temperature, cellular metabolism is suspended and cryopreserved cells can recover with maintained viability and functionality upon warming in a water bath at 37 °C.

Numerous cryoprotectants have been investigated for the cryopreservation of MSCs, with the aim of reducing the extracellular ice formation, preventing excessive solute concentration, or minimizing cell dehydration. The cryoprotectants are classified into two categories: permeating and non-permeating, based on their ability to cross the cell membrane. Examples of permeating cryoprotectants include DMSO, ethylene glycol, and glycerol, while non-permeating cryoprotectants include trehalose, sucrose, and sorbitol [7,8,9]. Conventionally, 5% or 10% DMSO is used with additives (e.g., serum) as cryoprotectants for MSCs cryopreservation. Serum is commonly used as an additive for cryopreservation of MSCs due to its ability to stabilize cell membranes, adjust osmotic pressures, and protect cells from free oxygen radicals [10,11,12,13,14].

However, DMSO can cause alterations of cellular and genetic characteristics to post-freeze/thaw cells, and animal serum might carry a potential risk of transmitting xenogeneic antigens and pathogens [15,16,17,18,19,20,21,22], which could limit its clinical application. To address these limitations, various alternative cryoprotectants have been explored [23,24,25]. It was reported that a DMSO- and serum-free cocktail solution, consisting of 0.05 M glucose, 0.05 M sucrose, and 1.5 M ethylene glycol, was developed as a promising option for the cryopreservation of MSCs [25].

Despite extensive studies on cryoprotectants, the issue of cytoskeletal damage (e.g., actin disruption) induced by freeze/thaw stress has not been fully addressed. Cytoskeletal dynamics, specifically actin polymerization and arrangement, plays an important role not only in cell survival and proliferation but also in homing/engraftment properties of MSCs. Consequently, actin disruption caused by freeze/thaw stress can have a significant impact on the efficacy of MSCs [26,27,28,29,30]. Therefore, the development of cryoprotectants that possess cryoprotective potential to minimize actin cytoskeletal damage is crucial for improving MSC cryopreservation.

This study aimed to evaluate the efficacy of the DMSO- and serum-free cocktail solution combined with an actin stabilizer, sphingosine-1-phosphate (S1P), for cryopreservation of DP-MSCs, with a focus on actin stabilization and cell viability. S1P is a bioactive sphingolipid known to play a crucial role in diverse biological processes. It acts as a signaling molecule and binds to specific G protein-coupled receptors known as S1P receptors (S1PR), which are located on the cell surface. S1P/S1PR signaling induces cell survival, proliferation, and differentiation, as well as cytoskeleton dynamics (e.g., actin polymerization) [31,32,33,34,35,36]. For this reason, S1P has garnered attention as an additive for cryopreservation, with some studies suggesting that S1P treatment could maintain viability and functionality of cryopreserved cells or tissues [37,38]. To evaluate the cryoprotective potential of S1P, this study assessed the safety of S1P treatment in DP-MSCs, and comparatively analyzed the cell survival rates, cell viability, actin cytoskeleton, and adhesion ability of freeze/thawed DP-MSCs with different cryoprotectants.

## 2. Materials and Methods

Unless otherwise specified, all chemicals and media used in this study were purchased from Sigma-Aldrich (St. Louis, MO, USA) and Gibco (Thermo Fisher Scientific, Inc., Waltham, MA, USA), respectively.

### 2.1. Preparation of DP-MSCs

Dental pulp-derived MSCs (DP-MSCs) were isolated from human dental pulp tissues, as previously described [39]. In brief, third molars were collected from donors aged 18.5 ± 2.3 years at the Department of Oral and Maxillofacial Surgery at Changwon Gyeongsang National University Hospital following approval by the Institutional Review Board of University Hospital, and with the informed consent of enrolled patients for their tissue donation (GNUH-IRB-2018-11-002-001). The dental pulp tissue was aseptically separated from the dental crown after fracture with bone forceps and washed with Dulbecco’s phosphate buffer saline (DPBS), which contained 1% penicillin-streptomycin (10,000 IU and 10,000 μg/mL, respectively, Pen-Strep). The tissue was then chopped into small pieces (approximately 1 mm^3^) using sterile scissors and treated with 1 mg/mL collagenase type I for 60 min at 37 °C to digest the extracellular matrix and release the cells. After digestion, to obtain a single-cell suspension, the cell suspensions were collected by gradually passing through a 100-μm and 40-μm cell strainers (Falcon^®^; Corning, Inc., New York, NY, USA), respectively. Finally, the collected cells were placed into 100 mm plastic culture dish containing advanced Dulbecco’s modified eagle medium (ADMEM) supplemented with 10% fetal bovine serum (FBS), 1% GlutaMAX and 1% Pen–Strep. The cells were cultured in 25T-flasks (Nunc™, Roskilde, Denmark) at 37 °C in a humidified atmosphere containing 5% CO_2_, and the medium was changed every 3 days. When the cells reached 80–90% confluence, they were passaged using 0.25% trypsin-EDTA to detach the cells from the dish and further expanded to increase and obtain the homogenous cell population. DP-MSCs were used for experiments at passage 3–5.

### 2.2. Characterization of DP-MSCs

To assess the marker expression of MSCs, cells were harvested using trypsinization and washed with DPBS. The cells were then incubated with fluorescently-labeled antibodies against the MSC markers CD44, CD73, CD90, and CD105, as well as antibodies against CD34, CD45, MHC class I, and MHC class II for 30 min at 4 °C in the dark and analyzed using flow cytometry. The data were analyzed using a standard flow cytometry software (BD FlowJo™ Software v10) to determine the percentage of cells expressing each marker.

To investigate differentiation potential to the mesenchymal lineage, DP-MSCs were differentiated into osteoblasts, adipocytes, and chondrocytes by following a previously published protocol [40]. In brief, the cells were cultured for 21 days in adipocyte (DMEM containing 10% FBS, 100 μM indomethacin, 10 μM insulin, and 1 μM dexamethasone), osteoblast (DMEM containing 10% FBS, 10 nM dexamethasone, 50 μg/mL ascorbic acid, and 10 mM sodium β-glycerophosphate), and chondrocyte (STEMPROTM Chondrogenesis Differentiation Kit) differentiation medium, respectively. Adipogenesis was confirmed by the formation of lipid droplets by staining with Oil red O solution. Osteogenesis was confirmed by the presence of calcium deposits by staining with Alizarin red. Chondrogenesis was confirmed by the presence of proteoglycans by staining with Alcian blue.

### 2.3. qRT-PCR

The relative mRNA expression levels of pluripotent markers (NANOG and OCT4) and apoptotic markers (BAK and BAX) were analyzed by qRT-PCR in triplicates. Total RNA from DP-MSCs treated with or without 10 μM S1P (Cayman chemical, Ann Arbor, MI, USA) in a culture medium for 1 h was extracted using the easy-spinTM Total RNA Extraction Kit (iNtRON Biotechnology, Seongnam, South Korea) according to the manufacturer’s instructions. The RNA was quantified using an OPTIZEN NANO Q spectrophotometer (Mecasys, Daejeon, South Korea), and cDNA was synthesized with 500 ng of RNA using HiSenScriptTM RH(-) RT PreMix Kit (iNtRON Biotechnology). qRT-PCR was performed using RealMODTM Green AP 5x qPCR mix (iNtRON Biotechnology) and specific primers for the genes. The PCR reaction cycle consisted of an initial activation at 95 °C for 12 min, followed by 40 cycles of PCR at 95 °C for 15 s, 60 °C for 25 s, and 72 °C for 25 s. The amplification curves, melting curves, and cycle threshold values (Ct values) were analyzed using the Rotor-Gene Q Series Software 2.1.0 (Qiagen, Helden, Germany). The Ct values were normalized to the expression level of the housekeeping gene, ACTB, and all samples were analyzed in triplicate to ensure reproducibility. The relative mRNA expression levels were calculated using the 2^−ΔΔCt^ method. The primers used in this study are listed in Table 1.

### 2.4. Cryopreservation and Thawing of MSCs

DP-MSCs were resuspended in 1 mL cryoprotectants at a concentration of 1 × 10^6^ cells/mL and transferred to 1.8 mL cryovials (Thermo Fisher Scientific). The cryovials were cooled at approximately −1 °C/min from 25 °C to −80 °C in a freezing container (Nalgene^®^ Mr. Frosty^®^ Cryo 1 °C Freezing Container, Thermo Fisher Scientific), and then immediately plunged into liquid nitrogen (LN2). Cells were divided into 5 experimental groups, as follows. Non-cryopreserved DP-MSCs (Fresh, control); DP-MSCs cryopreserved by conventional method with 10% DMSO and 10% FBS in ADMEM (DMSO); DP-MSCs cryopreserved with cocktail solution containing 0.05 M glucose, 0.05 M sucrose, and 1.5 M ethylene glycol in ADMEM (Cocktail) by following previously published protocol (ref); DP-MSCs cryopreserved with cocktail solution by S1P pretreatment for 1 h before freezing (S1P→C); and DP-MSCs cryopreserved with the S1P-added cocktail solution (C + S1P). The cryopreserved DP-MSCs were thawed in a circulating water bath at 37 °C for 1 min and washed with the culture medium to remove cryoprotectants. For analysis of cell survival rate, the cells immediately stained with 0.4% trypan blue and percentage of the viable cells excluding trypan blue were counted by Countess Automated Cell Counter (Invitrogen, Carlsbad, CA, USA). For further analysis, the DP-MSCs of all groups were seeded at in culture plates and cultured for 24 h.

### 2.5. Cell Viability and Proliferation Analysis

To investigate the viability of DP-MSCs after S1P treatment, the WST-1 assay was performed according to the manufacturer’s protocol. Briefly, DP-MSCs were seeded in 96-well plates at a density of 5 × 10^3^ cells/well and cultured for 24 h. Cells were then treated with or without 10 μM S1P. After 1 h of treatment, WST-1 reagent (Abcam, #ab65475) was added to each well, and the cells were incubated for 2 h at 37 °C. The absorbance was measured at 450 nm using a VersaMax™ Tunable Microplate Reader (Molecular Devices, San Jose, CA, USA).

To evaluate the rates of early and late apoptosis in the fresh or post-freeze/thaw DP-MSCs, Annexin V-propidium iodide (PI) assay was performed according to the manufacturer’s instructions of Dead Cell Apoptosis Kits (Invitrogen, #V13242). In brief, cells were seeded in 24-well cell culture plates (the density of 5 × 10^4^ cells/well). The harvested cells at 24 h of culture were stained with Annexin V-FITC (50 μL/mL and 100 μL/mL, and the cells were immediately analyzed using FACSVerse™ flow cytometer (BD Biosciences, Franklin Lakes, NJ, USA) and FlowJo 10.1.0 software. At least 10,000 cells were counted for each sample.

Population doubling time (PDT) was used to investigate cell cycle time for proliferation. Briefly, the fresh or post-freeze/thaw DP-MSCs were plated at 24-well culture plate in triplicate (the density of 2 × 10^3^ cells/well) and then cultured for 7 days. The cell number was counted by hemocytometer. The PDT of DP-MSCs was calculated using the following formula: PDT = t (log2)/(logNt − logN0), where t = the culture time, N0 = the number of cells initially at time 0, and Nt = the number of cells at time t.

### 2.6. F-Actin Staining

The fresh or post-freeze/thaw DP-MSCs were seeded on 12 mm glass coverslips (SPL Life Sciences, Pocheon, South Korea) and then cultured in an incubator (at 37 °C for 24 h). The cells were fixed with 4% paraformaldehyde in PBS for 15 min at room temperature and further permeabilized in 0.1% Triton™ X-100 in PBS for 15 min. After being washed in PBS, the fixed cells were stained with Alexa Fluor™ 488 Phalloidin (Invitrogen) according to the manufacturer’s instructions. For mounting, the coverslips with the cells were treated with VECTASHIELD^®^ Antifade Mounting Medium (Vector Laboratories, Newark, CA, USA) and then placed on slides. The stained cells were imaged with a Nikon Eclipse Ti-U (Nikon Corporation, Tokyo, Japan). The number of disrupted cells with actin cytoskeletal disruption and unstable morphology was manually counted along with the total number of cells. The percentage of actin disruption was calculated as the number of disrupted cells divided by the total number of cells multiplied by 100.

### 2.7. Cell Adhesion Assay

The fresh or post-freeze/thaw DP-MSCs were seeded in a 6-well cell culture plate (the density of 1 × 10^5^ cells/well) and cultured for 4 h. After incubation, the culture medium with non-adherent cells was collected and centrifuged (at 350× *g* for 5 min). The number of non-adherent cells was manually counted using hemocytometer. The percentage of cell adhesion was calculated by the formula, (the number of seeded cells—the number of non-adherent cells) × 100/the number of seeded cells.

### 2.8. Statistical Analysis

All experimental data were analyzed using GraphPad Prism version 8. The statistical differences were analyzed by *t*-test and one-way analysis of variance (ANOVA) followed by Tukey’s multiple comparisons test. The data are presented as mean ± standard deviation (SD), and statistical significance was defined as *p* < 0.05.

## 3. Results

### 3.1. Characterization of DP-MSCs by Surface Markers Expression and Differentiation Potential

**Figure 1:**
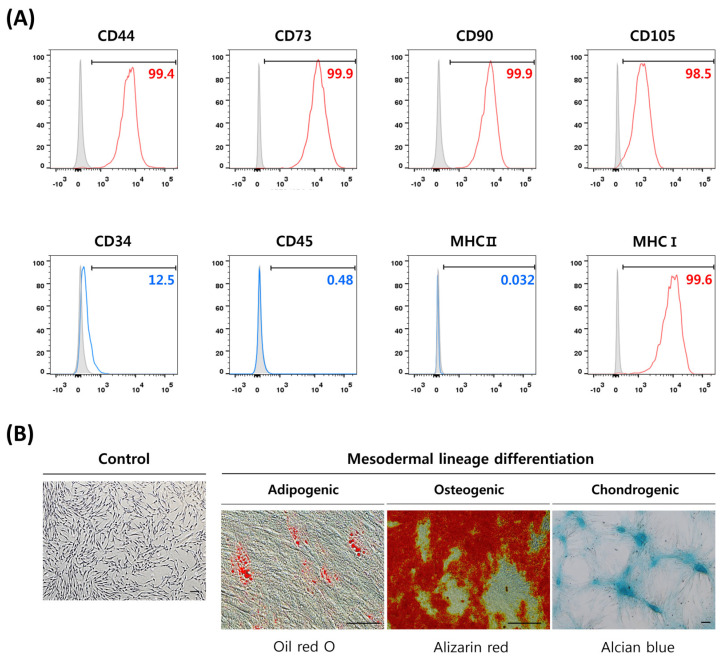
Characterization of mesenchymal stem cells (MSCs) by cell surface marker expression and differentiation potential. MSCs were analyzed for the expression of various markers by flow cytometry, and their ability to differentiate into adipocytes, osteoblasts, and chondrocytes was assessed by positive expression of relevant stains. (**A**) MSCs were found to express CD44, CD73, CD90, CD105, and MHC class I, consistent with MSCs identity. MSCs also lacked expression for CD34, CD45, and MHC class II, which are not typical of MSCs. (**B**) MSCs exhibited mesodermal lineage differentiation potential, as shown by staining for adipogenesis (Oil red O), osteogenesis (Alizarin red), and chondrogenesis (Alcian blue). Scale bar is 100 μm.

DP-MSCs isolated from human dental pulp were characterized by their cell surface markers expression of MSCs and their differentiation potential into mesodermal lineages. Flow cytometric analysis revealed the MSCs phenotype of the isolated DP-MSCs, with high expression of positive MSCs markers (CD44, CD73, CD90, CD105, and MHC class I) and low-to-no expression of negative markers (CD34, CD45, and MHC class II) (Figure 1A). The mesodermal lineage differentiation potential of DP-MSCs was evaluated through the induction of adipocyte, osteoblast, and chondrocyte differentiation using lineage specific induction media. The differentiated cells were then characterized by staining for lipid droplets in adipocytes with Oil red O, calcium deposition in osteoblasts with Alizarin red, and proteoglycan production in chondrocytes with Alcian blue (Figure 1B). All the MSC groups were successfully differentiated into mesodermal lineages and displayed a positive staining expression for all the relevant stains. The successful induction of differentiation and characterization of the DP-MSCs confirmed their mesenchymal stem cell characteristics.

### 3.2. S1P Treatment Does Not Induce a Negative Impact on the Viability and Stemness of DP-MSCs

**Figure 2:**
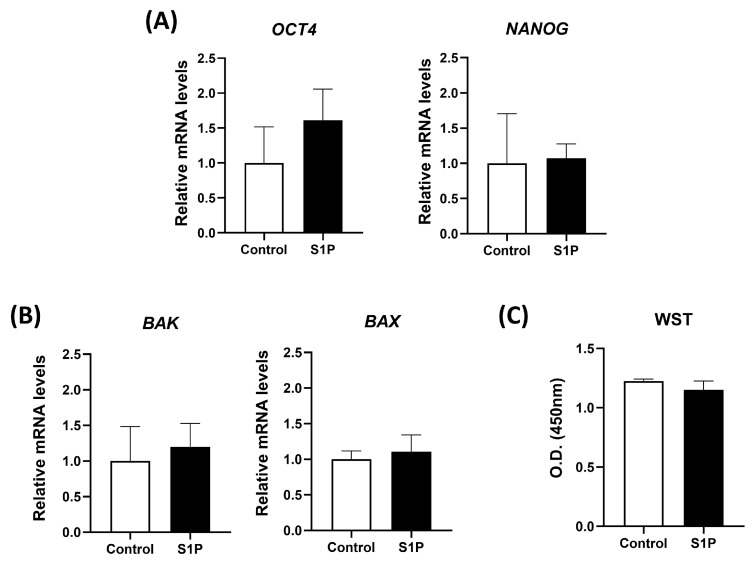
Effects of S1P treatment on mRNA relative levels of pluripotent and apoptotic markers, and cell viability in DP-MSCs. (**A**,**B**) Relative mRNA levels of pluripotent markers (NANOG and OCT4) and apoptotic markers (BAK and BAX) in DP-MSCs were analyzed by RT-qPCR after treatment with S1P (10 μM) for 1 h. S1P treatment had no significant effect on the mRNA levels of NANOG, OCT4, BAK and BAX. (**C**) The effect of S1P treatment on cell viability was assessed using the WST-1 assay. S1P treatment had no effect on cell viability. Data are presented as mean ± SD (*n* = 3). Statistical significance was determined using *t*-test compared to the control (non-treated DP-MSCs).

To evaluate the safety of S1P treatment on DP-MSCs, the relative mRNA expression levels of pluripotent markers (NANOG and OCT4) and apoptotic markers (BAK and BAX) were measured after exposing DP-MSCs to S1P for 1 h. S1P treatment did not differ the expression levels of NANOG, OCT4, BAK, and BAX compared to control (Figure 2A,B). The WST-1 assay was performed to analyze cell viability after S1P treatment for 24 h; we found no negative impact on cell viability (Figure 2C). These results provide compelling evidence that S1P treatment is safe for DP-MSCs.

### 3.3. S1P Pretreatment Enhanced the Cell Viability of Post-Freeze/Thaw DP-MSCs

**Figure 3:**
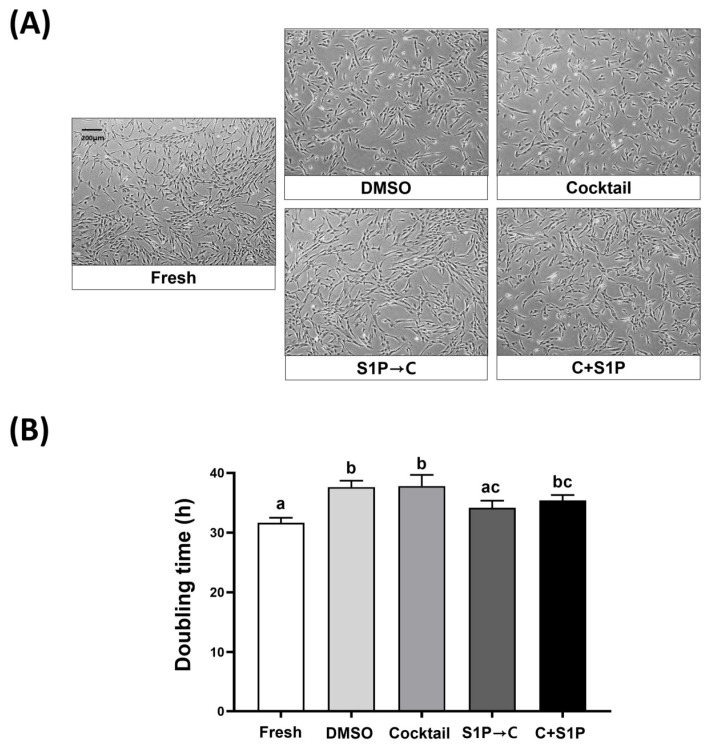
Proliferation properties of post-freeze/thaw DP-MSCs. (**A**) Morphology of DP-MSCs at 3 days after thawing and culturing on culture plate (scale bar, 200 μm; magnification, ×40). (**B**) Proliferation properties of DP-MSCs at 24 h after thawing and culture, by calculating PDT. DP-MSCs cryopreserved with S1P→C demonstrated significantly (*p* < 0.05) enhanced proliferation properties with lower PDT compared to those cryopreserved with DMSO and Cocktail. Data represent mean ± SD of three independent experiments; different letters (a–c) denote statistical differences between groups (*p* < 0.05).

To evaluate the cryoprotective potential of the different methods, the viability and morphology of DP-MSCs were analyzed after thawing and culturing for 24 h. The spindle fibroblast-like morphology was observed in DP-MSCs of all groups, indicating no significant changes in cell shape due to the cryopreservation process. After three days of culture, DP-MSCs cryopreserved with S1P→C and C + S1P showed higher confluency compared with those cryopreserved with DMSO and Cocktail (Figure 3A). The doubling time of the fresh or post-freeze/thaw DP-MSCs was calculated, and the results showed that DP-MSCs cryopreserved with DMSO (37.63 ± 1.1%), Cocktail (37.8 ± 1.9%), C + S1P (35.4 ± 0.9%) showed significantly (*p* < 0.05) longer doubling times compared to those in control (31.7 ± 0.8%). However, the doubling times of DP-MSCs cryopreserved with S1P→C (34.2 ± 1.2%) did not differ significantly from that in the control and was significantly (*p* < 0.05) shorter than that in DMSO and Cocktail (Figure 3B).

**Figure 4:**
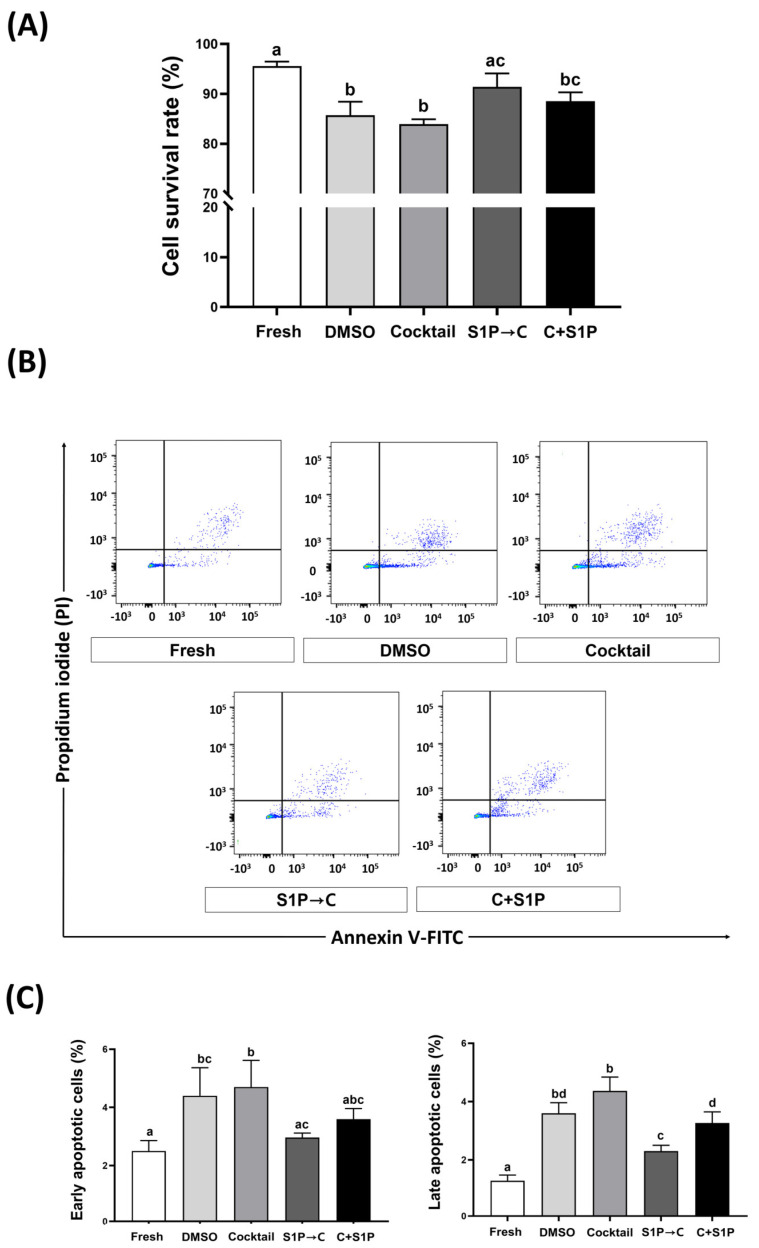
Cell viability of post-freeze/thaw DP-MSCs. (**A**) Cell survival rate of DP-MSCs at 0 h after thawing, using count of un-stained cells in trypan blue staining. (**B**,**C**) For apoptosis, DP-MSCs were stained with Annexin V-FITC/PI and analyzed by flow cytometry at 24 h after thawing and culture. DP-MSCs cryopreserved with S1P→C demonstrated significantly (*p* < 0.05) enhanced viability with higher survival rate and lower apoptosis compared to those cryopreserved with DMSO and Cocktail. Data represent mean ± SD of three independent experiments; different letters (a–d) denote statistical differences between groups (*p* < 0.05).

The survival rate of post-freeze/thaw DP-MSCs in DMSO (85.7 ± 2.8%), Cocktail (84.0 ± 1.0%), and C + S1P (88.5 ± 1.8%) was measured using trypan blue staining and found to be significantly lower than that in control (95.6 ± 0.9%). However, the survival rate of DP-MSCs cryopreserved with S1P→C (91.4 ± 2.7%) did not significantly differ from was that in control and was significantly (*p* < 0.05) higher than that in DMSO and Cocktail (Figure 4A). After 24 h of post-freeze/thaw culture of DP-MSCs, the percentage of early and late apoptotic cells in DP-MSCs was measured using flow cytometry, following staining with Annexin V-FITC and PI. The percentage of early apoptotic cells (Annexin V+/PI−) in DMSO (4.4 ± 1.0%) and Cocktail (4.7 ± 0.9%), were significantly (*p* < 0.05) higher than that in control (2.5 ± 0.4%). However, the percentage of early apoptotic cells in S1P→C (3.0 ± 0.2%) and C + S1P (3.6 ± 0.4%) did not significantly differ from that in the control. The percentage of late apoptotic cells (Annexin+/PI+) in all cryopreserved groups (DMSO, 3.6 ± 0.4%; Cocktail, 4.4 ± 0.5%; S1P→C, 2.3 ± 0.2%, and C + S1P, 3.3 ± 0.4%) was significantly (*p* < 0.05) higher than that in control (1.3 ± 0.2%). Nonetheless, the percentage of late apoptotic cells in DP-MSCs cryopreserved with S1P→C was significantly (*p* < 0.05) lower than those of DP-MSCs cryopreserved with DMSO and Cocktail (Figure 4B,C).

### 3.4. S1P Pre-Treatment Reduced Actin Cytoskeletal Disruption and Improved Adhesion Ability

**Figure 5:**
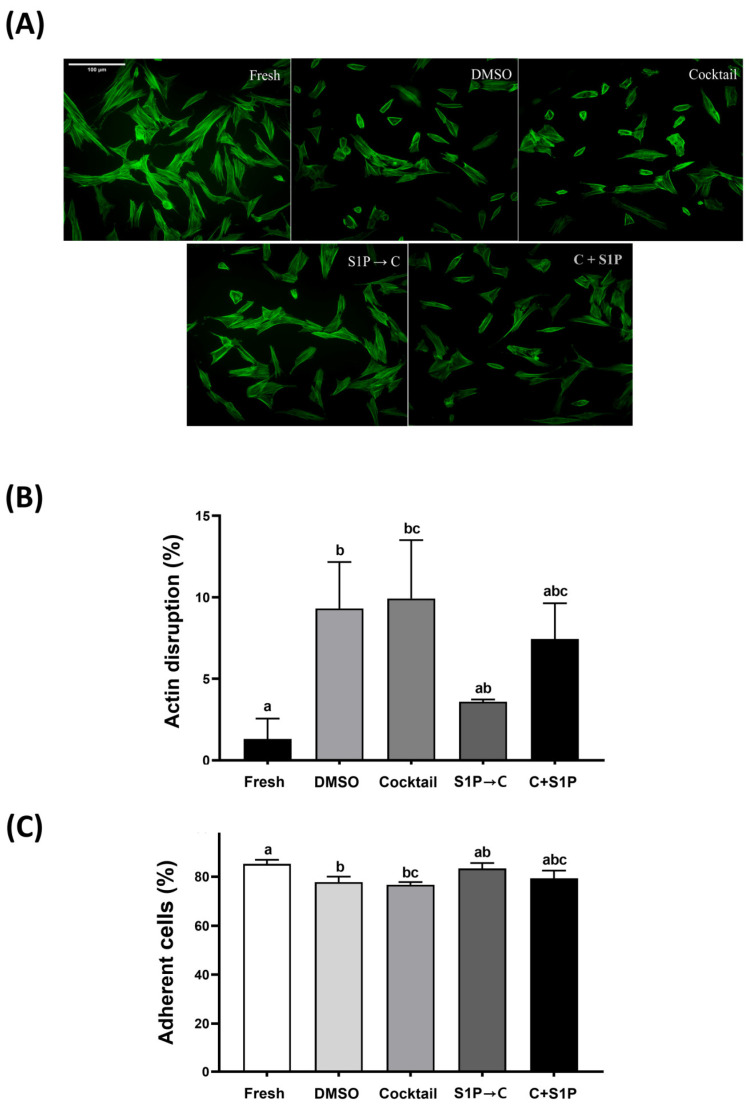
Actin disruption and adhesion ability of post-freeze/thaw DP-MSCs. (**A**,**B**) F-actin in DP-MSCs stained with phalloidin (Green, conjugated with Alexa 488) at 24 h after thawing and culture (scale bar = 100 μm; magnification = ×100). (**C**) Adhesion ability of DP-MSCs by calculating the non-adherent cells at 24 h after thawing and seeding on culture plate. DP-MSCs cryopreserved with S1P→C exhibited a significantly (*p* < 0.05) higher adhesion ability, with lower levels of actin disruption, compared to those cryopreserved with DMSO and Cocktail. (**A**–**C**) Data represent ± SD of three independent experiments; different letters (a–c) denote statistical differences between groups (*p* < 0.05).

F-actin staining was performed using Alexa Fluor™ 488 Phalloidin to evaluate the change in actin cytoskeleton of the fresh or post-freeze/thaw DP-MSCs. DP-MSCs in all groups showed disrupted cells, defined as cells with visible actin disruption and unstable morphology (Figure 5A). DP-MSCs cryopreserved with DMSO (9.3 ± 2.8%) and Cocktail (9.9 ± 3.6%) showed a significant (*p* < 0.05) increase of actin disruption, compared to that in control (1.3 ± 1.3%). However, DP-MSCs cryopreserved with S1P→C (3.6 ± 0.1%) showed a significantly (*p* < 0.05) lower percentage of actin disruption, compared to that in Cocktail (Figure 5B). In other words, the variation in the number of cells with respect to actin disruption was seen among different groups. Furthermore, the adhesion ability was evaluated by cell adhesion rate after post-freeze/thaw culture. DP-MSCs cryopreserved with DMSO (77.8 ± 2.2%), Cocktail (76.7 ± 1.1%) showed a significant (*p* < 0.05) decrease in adhesion ability compared to that in control (85.2 ± 1.7%). However, DP-MSCs cryopreserved with S1P→C had a significantly (*p* < 0.05) higher adhesion rate (83.3 ± 2.3%) compared to that in Cocktail, and no significant difference compared to that in control (Figure 5C). Overall results indicate that S1P possesses a cytoprotective role among various parameters and is proven to be safer for DP-MSCs.

## 4. Discussion

The main purpose of this study was to develop a cryopreservation method for DP-MSCs that enhances cryoprotective potential and facilitates the clinical application. Cryoreservation is an essential method for the long-term preservation of stem cells, ongoing research is focused on its successful clinical application [41]. A number of cryopreservation methods have been reported to preserve different source derived MSCs and gradual efforts have been made to improve the development and use of cryoprotectants. In previous studies, MSCs derived from various tissues such as adipose, bone marrow, and dental pulp were cryopreserved, and their survival rates were compared after thawing. After undergoing cryopreservation and thawing, adipose tissue-derived MSCs did not show a significant change in survival rate, whereas the survival rates of bone marrow- and dental pulp-derived MSCs significantly decreased [11]. This indicates that cryopreservation methods for MSCs should be optimized depending on the tissue source. Some researchers have reported studies on cryoprotectants for cryopreservation of DP-MSCs [42,43,44,45]. Woods et al. (2009) compared DP-MSCs cryopreserved with various cryoprotectants at different concentrations (0.5–1.5 M) and reported that the highest survival rates (90.6 ± 8.9% and 91.0 ± 9.1%) were observed in 1 M and 1.5 M DMSO. However, as DMSO has potential toxicity (e.g., genetic alteration), it is important to develop new cryoprotectants that do not use DMSO, so that MSCs can be safely used for clinical application of DP-MSCs [42]. Lin et al. (2015) proposed “static magnetic field” as a cryopreservation method that replaces DMSO in DP-MSCs. However, there was a difference in the survival rate before and after the cryopreservation of DP-MSCs using the static magnetic field, but their survival rates were lower than that of cryopreservation using DMSO [44]. In other studies, researchers reported the use of DMSO- and serum-free cocktail solutions as cryoprotectants for cryopreservation of human dental tissue [46] and Wharton’s Jelly-derived MSCs (WJ-MSCs) [25].

MSCs have abundant potential as cell therapeutics in regenerative medicine because of their homing/engraftment ability, as well as therapeutic efficacy through regenerative and immunomodulatory abilities [47,48,49]. In the context of these abilities of MSCs, cytoskeleton is an absolute necessity. Cytoskeleton, including actin filaments, plays an important role in changing the cell shape, repositioning internal organelles, and migrating to other places, which can occur through the modification of the cell shape according to the assembly and disassembly of microfilaments and microtubules [50,51]. Although some studies have reported the occurrence of actin cytoskeletal disruption during cryopreservation for long-term storage of MSCs [26,29], there is a lack of research exploring cryopreservation methods to stabilize the actin cytoskeleton of cryopreserved MSCs. In this study, we used S1P to stabilize the actin cytoskeleton in a freeze/thaw stress environment. It has been reported that S1P can regulate actin cytoskeleton, which is known to contribute to improving cell proliferation, differentiation, and migration [50]. In accordance, this study aimed to develop f a new cryopreservation method that enhances cryoprotective potential by verifying the safety of S1P on cells and minimizing actin disruption that can occur during the freeze/thaw process.

In this study, DP-MSCs were isolated from dental pulps in fresh human dental tissues and successfully characterized through the expression of specific surface markers and mesodermal lineage differentiation potential. First, the safety of S1P treatment in successfully isolated fresh DP-MSCs was investigated. 10 μM S1P treatment for 1 h did not significantly affect the expression of pluripotency and apoptosis markers and showed no negative impact on cell viability in WST-1 assay. These results suggest that S1P is safe to use for the cryopreservation of DP-MSCs. Romani et al. (2018) compared the effects of various concentrations of S1P treatment (0.01, 0.1, and 1 μM) on amniotic fluid-derived MSCs for 24 h. Cell visibility was significantly improved only at 0.01 μM S1P, as shown by BrdU and MTT assays. The expression of pluripotent markers such as OCT4 was significantly reduced at all concentrations [52]. Chen et al. (2018) showed that 0.3 μM S1P treatment for 24 h reduced the apoptotic cell rate of adipose tissue-derived MSCs subjected to oxidative stress induced by H_2_O_2_ [53]. Since this study aimed to establish the pretreatment conditions of S1P for cryopreservation of DP-MSCs, a higher concentration (10 μM) of S1P was used for a shorter period (1 h) compared to other studies. Therefore, the differences in results may have occurred due to different concentration and processing time of S1P treatment, and cell origins.

Cell viability is an important measure of cryoprotective potential. In this study, the cell viability of post-freeze/thaw DP-MSCs was found to be similar in both the 10% DMSO solution and the cocktail solution, and both were significantly lower than fresh DP-MSCs, which is similar to a previous study that used the cocktail solution for cryopreservation of WJ-MSCs [25]. However, in the study where dental tissue was cryopreserved, the cell viability of DP-MSCs isolated from human dental pulp tissue showed a significant difference (*p* < 0.05) between those cryopreserved in 10% DMSO solution (35.3 ± 4.2%) and in the cocktail solution (79.3 ± 2.5%). In addition, the proliferation capacity of DP-MSCs isolated from cryopreserved dental tissue using the cocktail solution was similar to that of DP-MSCs isolated from fresh dental tissue [46]. These differences in results suggest that the cryoprotective potential of 10% DMSO solution is more effective in DP-MSCs than in dental tissue, and the cocktail solution has effective cryoprotective potential in both tissue and cells but can reduce proliferation capacity in cells. Onions et al. (2008) reported that the use of a cryoprotectant containing 20 μM S1P did not have a beneficial effect on cell survival and proliferation in cryopreserved ovarian tissues [38]. Similarly, this study showed that a cryoprotectant containing 10 μM S1P had no difference in cell viability and proliferation in cryopreserved DP-MSCs, except for a decrease in late apoptosis rate. However, DP-MSCs, cryopreserved with the cocktail solution after S1P pretreatment, had significantly higher cell viability compared to than those in the cocktail solutions without S1P pretreatment. Although S1P has been reported to have anti-apoptosis effects as well as actin cytoskeletal stabilization potential, the result suggests that S1P pretreatment increases the cell viability of post-freeze/thaw DP-MSCs rather than S1P addition.

Rogoonanan et al. (2010) reported that freezing-induced dehydration in human foreskin fibroblasts can lead to a hyperosmotic environment, which causes to alteration of the actin cytoskeleton and cell membrane [27]. Similarly, in other studies, it was reported that freeze/thaw stress can impair actin polymerization and distribution in cryopreserved MSCs in a cryoprotectant containing 10% DMSO, which can reduce the adhesion ability of MSCs and potentially impact their therapeutic efficacy. Our current study results demonstrated the unstable actin cytoskeletal morphology of post-freeze/thaw DP-MSCs in both 10% DMSO solution and cocktail solution, as reported in previous studies [26,29]. On the other hand, it was shown that S1P pretreatment for cryopreservation of DP-MSCs reduces the alteration of the actin cytoskeleton and maintains adhesion ability at a level similar to that in fresh DP-MSCs. The results are consistent with the fact that actin filaments are important for adhesion ability and shape maintenance. Therefore, it was confirmed that S1P pretreatment can be an attractive method to overcome the effects of freeze/thaw stress on the actin cytoskeleton during cryopreservation. However, further investigation is required to determine the clear mechanisms for maintaining of actin cytoskeleton.

Along with cell viability, maintenance of pluripotent markers (stemness) and low-profile apoptosis status (safety) of targeted MSCs are also equally important evaluation parameters when dealing with any chemical/agent to be directly used in cells. In this regard, we investigated the cryoprotective effects of S1P with special focus on stemness and apoptotic status on DP-MSCs during cryopreservation. As expected, S1P treatment did not have a negative impact on cell viability or the relative mRNA expression levels of pluripotent and apoptotic markers, suggesting that S1P treatment is safe in DP-MSCs. Overall results indicate that S1P pretreatment before cryopreservation showed higher cryoprotective effects (e.g., improvement of cell viability, stemness maintenance, actin cytoskeleton, and adhesion ability) than using a cryoprotectant containing S1P. These findings provide a potential strategy for improving the quality and yield of DP-MSCs for clinical applications. However, further studies are needed to explore the underlying mechanisms of the observed effects and optimize the S1P treatment protocol. In summary, the results of this study suggest that that S1P treatment before cryopreservation can be used as a useful tool for preserving important stemness features of DP-MSCs so that their suitability in clinical applications can be enhanced.

## Figures and Tables

**Table 1:** List of primers used for the evaluation of transcription factors, apoptosis-related genes, and in cultured DP-MSCs by RT-PCR.

Gene	Primer Sequence	Product Size (bp)	Accession No.
OCT4	F: AAGCAGCGACTATGCACAAC R: AGTACAGTGCAGTGAAGTGAGG	140	NM_002701.5
NANOG	F: GCAGATGCAAGAACTCTCCAAC R: CTGCGTCACACCATTGCTATTC	133	AB093576.1
BAX	F: TCTGACGGCAACTTCAACTG R: AGTCCAATGTCCAGCCCATG	127	NM_001291428.1
BAK	F: GGCACCTCAACATTGCATGG R: CAGTCTCTTGCCTCCCCAAG	144	NM_001188.3
GAPDH	F: AGTCAGCCGCATCTTCTTTT R: CCAATACGACCAAATCCGTT	97	NM_002046.5

OCT4, octamer-binding transcription factor 4; BAX, Bcl-2-associated X protein; BAK, Bcl2-antagonist/killer.

## Data Availability

Not applicable.
